# Correction: Radioprotective effects of ultra-small citrate-stabilized cerium oxide nanoparticles *in vitro* and *in vivo*

**DOI:** 10.1039/d6ra90135b

**Published:** 2026-10-02

**Authors:** A. L. Popov, S. I. Zaichkina, N. R. Popova, O. M. Rozanova, S. P. Romanchenko, O. S. Ivanova, A. A. Smirnov, E. V. Mironova, I. I. Selezneva, V. K. Ivanov

**Affiliations:** a Institute of Theoretical and Experimental Biophysics, Russian Academy of Sciences Institutskaya 3 Pushchino Moscow region 142290 Russia antonpopovleonid@gmail.com +7 4967 739148; b Pushchino State Institute of Natural Sciences Pushchino Moscow region 142290 Russia; c Kurnakov Institute of General and Inorganic Chemistry, Russian Academy of Sciences Moscow Russia; d National Research Tomsk State University Russia

## Abstract

Correction for ‘Radioprotective effects of ultra-small citrate-stabilized cerium oxide nanoparticles *in vitro* and *in vivo*’ by A. L. Popov *et al.*, *RSC Adv.*, 2016, **6**, 106141–106149, https://doi.org/10.1039/c6ra18566e.

The authors regret an error in the scale bar of Fig. S2 and inconsistency in the reported nanoparticle size between this article and their previous article (ref. 1 below).

The authors confirm that the error in the scale bar of Fig. S2 did not affect any statement in the paper. The corrected Fig. S2 is shown below and the supplementary information (SI) has been updated.

Fig. S2 of this article demonstrates that the particle size distribution is quite broad, with the largest particles (4.5 nm) three- or four-fold greater than the smallest (∼1 nm). The average particle size was estimated by different people for this paper and ref. 1. Due to the broad particle size distribution and relatively low magnification of the image, the use of different calculation methods yielded different values. Neither this paper nor ref. 1 aimed at exact particle size calculation and the particle size value reported in this article was approximate. The authors are therefore confident that this discrepancy does not impact the conclusions of this work.


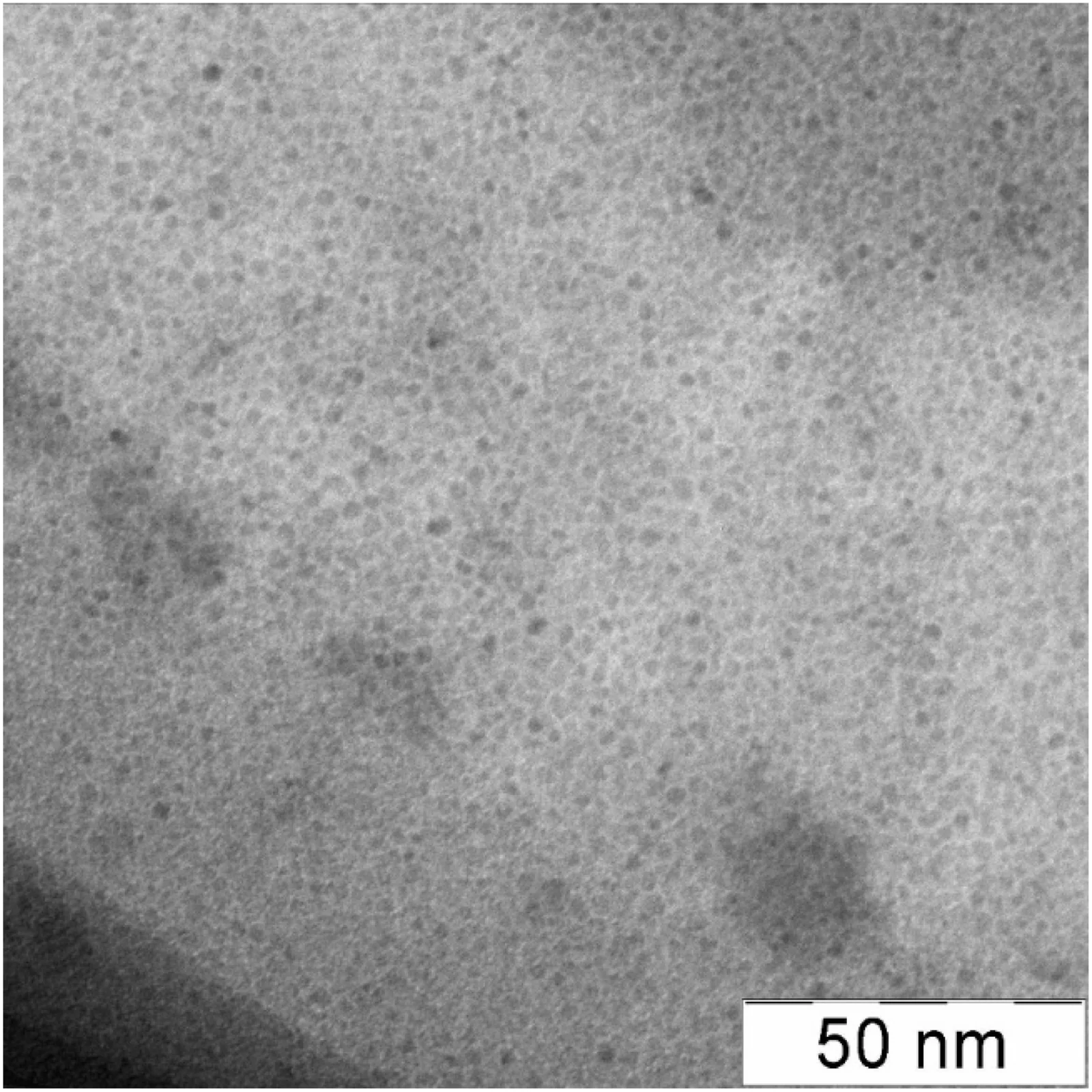
 **Fig. S2** TEM of citrate stabilized cerium oxide nanoparticles.

The Royal Society of Chemistry apologises for these errors and any consequent inconvenience to authors and readers.
